# Myocardial injury associated with naturally occurring clinical bovine anaplasmosis: evaluation of novel cardiac biomarkers

**DOI:** 10.1007/s11259-026-11309-x

**Published:** 2026-06-04

**Authors:** Şükrü Değirmençay, Sefa Küçükler, Selçuk Özdemir, Ramazan Kaman, Özge Kandemir, Reyhane Bayat, Muhammed Kadak, İrem Nur Göl, Nergis Ulaş, Rabia Yüksel, Akın Kırbaş

**Affiliations:** 1 Department of Internal Medicine, Faculty of Veterinary Medicine, Atatürk University, Erzurum, 25240 Turkey; 2 Department of Biochemistry, Faculty of Veterinary Medicine, Atatürk University, Erzurum, 25240 Turkey; 3 Department of Genetics, Faculty of Veterinary Medicine, Atatürk University, Erzurum, 25240 Turkey; 4 DVM Graduate, Veterinary Clinic, Kayseri, 38400 Turkey; 5 Aksaray Technical Sciences Vocational School, Aksaray University, Aksaray, 68100 Turkey; 6 Department of Veterinary Internal Medicine, Institute of Health Sciences, Atatürk University, Erzurum, Turkey

**Keywords:** Cattle, Anaplasmosis, Anaemia, Cardiac biomarkers, H-FABP, NT-proBNP

## Abstract

Severe anaemia and tissue hypoxia in bovine anaplasmosis may result in myocardial injury; however, the extent of cardiac involvement has not been comprehensively investigated using circulating cardiac biomarkers. The present study aimed to evaluate myocardial injury in cattle naturally infected with *Anaplasma* spp. and to determine the most reliable cardiac biomarkers for detecting cardiac damage. A case–control study was conducted in 24 Simmental calves, including 12 clinically affected calves and 12 healthy controls. Only clinically affected animals were included in the infected group, whereas quantitative real-time PCR *(*qPCR) positive but clinically normal cattle were excluded. Diagnosis was based on clinical examination, microscopic blood smear evaluation, and qPCR. Serum cTnI, CK-MB, AST, and LDH were measured using commercial enzyme kits, whereas H-FABP and NT-proBNP concentrations were determined using bovine-specific ELISA kits. Mean serum H-FABP (0.64 vs. 0.43 ng/mL; *P <* 0.001) and NT-proBNP (0.36 vs. 0.21 ng/mL; *P <* 0.001) concentrations, as well as median cTnI levels (0.11 vs. 0.06 ng/mL; *P =* 0.003), were significantly higher in cattle with anaplasmosis than in healthy controls. Haematocrit showed moderate negative correlations with H-FABP (*r =* -0.413, *P =* 0.045) and NT-proBNP (*r =* -0.617, *P =* 0.001). Heart rate showed strong positive correlations with H-FABP (*r* = 0.803, *P* < 0.001) and NT-proBNP (*r* = 0.732, *P* < 0.001), and a moderate positive correlation with cTnI (*r* = 0.537, *P* = 0.007). ROC analysis identified H-FABP as the best-performing biomarker (AUC = 1.000), followed by NT-proBNP (AUC = 0.903) and cTnI (AUC = 0.854). In conclusion, naturally occurring bovine anaplasmosis is associated with significant myocardial injury likely related to anaemia-induced hypoxia and compensatory tachycardia. Among the evaluated biomarkers, H-FABP demonstrated the highest diagnostic performance and may facilitate earlier recognition of cardiac involvement in affected cattle.

**Supplementary Information:**

The online version contains supplementary material available at https://doi.org/10.1007/s11259-026-11309-x.

## Introduction

Bovine anaplasmosis is an infectious, noncontagious, tick-borne rickettsial disease caused by obligate intra-erythrocytic bacteria of the genus *Anaplasma* (Radostitis et al. 2000; Inokuma 2007). The causative agents in cattle are *Anaplasma marginale* and *A. centrale*. Among these, *A. marginale* is the predominant and highly pathogenic species, inducing fever, haemolytic anaemia, jaundice, reduced milk production, anorexia, depression, abortion, and potentially death, whereas *A. centrale* typically causes mild anaemia. Following erythrocyte invasion and replication, infected cells are removed by the reticuloendothelial system, leading to haemolytic anaemia and icterus (Potgieter and Stoltsz 2004; Kocan et al. 2010; Hove et al. 2018). These changes result in systemic hypoxia and may induce degenerative lesions in multiple organs, including myocardial degeneration and coagulation necrosis (Jaswal et al. 2015; Das et al. 2021).

Despite histopathological evidence of cardiac involvement, biochemical evaluation of myocardial injury in bovine anaplasmosis remains limited. In contrast, similar haemoparasitic diseases such as theileriosis, which also induce anaemia and hypoxia (Temiz et al. 2014), have been associated with significant increases in cardiac biomarkers including cardiac troponin I (cTnI), creatine kinase myocardial band (CK-MB), and aspartate aminotransferase (AST) (Fartashvand et al. 2013). Conventionally, cTnI, CK-MB, creatine kinase (CK), AST, and lactate dehydrogenase (LDH) are used to assess myocardial injury, although some lack cardiac specificity.

Recently, new parameters indicating early heart damage have been found. Heart-type fatty acid-binding protein (H-FABP) is a low-molecular-weight fatty acid-carrying protein found in cardiomyocyte cytoplasm. As a cytoplasmic protein that plays a role in mitochondrial beta-oxidation, it constitutes 10% of cardiac myocyte cytosolic proteins (Başar et al. 2013). H-FABP is a new cardiac marker used in the early (first two hours) diagnosis of acute myocardial infarction (MI) and myocyte damage. Studies in humans have reported that H-FABP is a better diagnostic marker for early diagnosis (≤ 6 h) with high sensitivity (79%) and specificity (93%) compared to CK-MB and cardiac troponin I (cTnI) (Gerede et al. 2013). However, due to its rapid clearance from circulation, H-FABP may be less informative in later stages of myocardial injury. Therefore, its diagnostic utility is considered complementary to cardiac troponins, which remain elevated for a longer duration and reflect ongoing structural myocardial damage (Kleine et al. 1992). In veterinary medicine, increased H-FABP concentrations have been reported in cattle with bovine respiratory disease complex and traumatic pericarditis, supporting its diagnostic utility (Yildiz et al. 2019; Değirmençay 2023).

Natriuretic peptides, including atrial natriuretic peptide (ANP) and B-type natriuretic peptide (BNP), are released in response to myocardial wall stress, volume overload, and hypoxia (Potter et al. 2009; van Kimmenade and Januzzi 2009). The ventricular myocardium releases BNP and NT-proBNP hormones in response to pressure and volume overload. While BNP is eliminated in the blood within 20 min, NT-proBNP remains high in the blood for up to 60–120 min (Hall 2004). Elevated NT-proBNP levels have been documented in cattle with sepsis, respiratory disease, and traumatic pericarditis (Beydilli and Gökçe 2020; Ayvazoğlu et al. 2023; Değirmençay 2023).

Cardiac troponins (cTnI and cTnT) are highly specific markers of myocardial injury and remain elevated as long as myocardial damage persists (O’Brien 2008). CK-MB is a heart-associated isoenzyme of creatine kinase that increases following myocardial damage, although it is less specific than troponins (Mellanby et al. 2009; Liu et al. 2024). Elevated cTnI and CK-MB concentrations have been reported in calves with sepsis (Beydilli and Gökçe 2020; Kirbas et al. 2021), supporting their clinical utility. The amount of cTn in the contractile apparatus is 13–15 times greater than the amount of CK-MB per gram of myocardium (Wells and Sleeper 2008). However, enzymes such as LDH, AST, and CK-MB may also originate from non-cardiac tissues, limiting their specificity when used alone (Aslani et al. 2013). These biomarkers have been widely applied in haemoparasitic diseases, including theileriosis and babesiosis, to assess cardiovascular involvement (Hasanpour et al. 2008; Fartashvand et al. 2013; Orunç Kilinç et al. 2015; Razavi et al. 2015; Değirmençay et al. 2021).

Given that bovine anaplasmosis is characterized by haemolytic anaemia and consequent tissue hypoxia, myocardial injury may occur secondary to reduced oxygen delivery and compensatory cardiovascular responses (Singh et al. 2014; Jaswal et al. 2015; Değirmençay et al. 2023). We hypothesized that the severity of anaemia in cattle naturally infected with *Anaplasma* spp. would be associated with increased myocardial injury, reflected by elevations in circulating cardiac biomarkers and accompanied by compensatory tachycardia. However, the relationship between anaemia severity and cardiac biomarker alterations has not been systematically investigated in cattle naturally infected with *Anaplasma* spp. Therefore, the present study aimed to evaluate myocardial injury associated with naturally occurring bovine anaplasmosis by assessing conventional and novel cardiac biomarkers. In addition, the study investigated whether differences in infecting *Anaplasma* spp. (*A. marginale*, *A. centrale*, or mixed infection) were associated with variations in haematological alterations and myocardial injury biomarkers.

## Material and method

### Study population and design

This study was designed as a case–control investigation conducted on clinically affected calves diagnosed with anaplasmosis and age-matched healthy controls. The animals included in the study were 10–12-month-old Simmental calves of both genders. Based on clinical findings, blood smear evaluation, complete blood count (CBC), and real-time PCR results, the animals were classified into two groups: anaplasmosis (infected, *n* = 12) and healthy controls (*n* = 12). Because young animals may be qPCR-positive yet clinically normal, only animals exhibiting clinical signs consistent with anaplasmosis (fever, jaundice/pale mucous membranes, anorexia, tachycardia, weakness) were included in the infected group, whereas cattle that were qPCR-positive but clinically normal were excluded. All control animals were confirmed negative for *Anaplasma* spp. by qPCR and *Babesia* spp. and *Theileria* spp. by RT-qPCR and blood smears to exclude subclinical hemoparasitic infections. All calves underwent thorough clinical examination, including assessments of rectal temperature (RT), heart rate (HR), respiration rate (RR), and mucous membrane colour. Screening for concurrent diseases was based on a combination of detailed clinical examination, complete blood count, and serum biochemical analysis, including liver and renal function parameters. No abnormalities consistent with respiratory disease, sepsis, nutritional myopathy, or gastrointestinal disorders were identified. All clinical examinations and blood sampling were performed at initial presentation, prior to the administration of any therapeutic intervention, including antimicrobial and supportive treatments. The animals originated from smallholder farms located in Eastern Anatolia (Erzurum province, Türkiye) and were presented to the Atatürk University Faculty of Veterinary Medicine Animal Hospital in February. Initial blood sampling was performed under field conditions prior to referral, and samples were subsequently processed at the laboratory. The study protocol was approved by the Atatürk University Animal Experiments Local Ethics Committee (2022/5).

### Blood sampling

Blood samples (about 10.0 mL) were taken from calves via venipuncture of the jugular vein into tubes with EDTA (Vacutainer, K2E 3.6 mg, BD, UK) and gel (Vacutainer, BD, UK). For hematologic analysis and microscopic examination, whole blood was utilized, and the blood without anticoagulant was allowed to clot before being centrifuged at 1734 x g for 10 min at 4 °C to extract serum, which was stored at −80 °C until the day of the biochemical analysis.

### Microscopic examination

To observe the intraerythrocytic forms of *Anaplasma* spp. and other potential blood parasites (*Theileria* spp. and *Babesia* spp.), thin blood smears were prepared from EDTA-treated blood samples. The use of EDTA blood was necessitated by field conditions that precluded immediate smear preparation from fresh samples at the sampling site. Although EDTA may reduce parasite visibility in some cases, microscopic examination in the present study was used primarily as a supportive diagnostic tool. Definitive identification of *Anaplasma* species and detection of co-infections were based on qPCR and RT-qPCR analysis, respectively, thereby minimizing potential diagnostic limitations associated with EDTA-based smear preparation. Smears were fixed with absolute methanol (5 min), stained with 10% Giemsa solution (45 min), and examined under oil immersion (x1000). Parasitaemia was semi-quantitatively assessed and expressed as the percentage of infected erythrocytes after evaluation of at least 100 microscopic fields.

### Molecular detection of *Anaplasma spp.* and hemoparasite co-infections

**Fig. 1:**
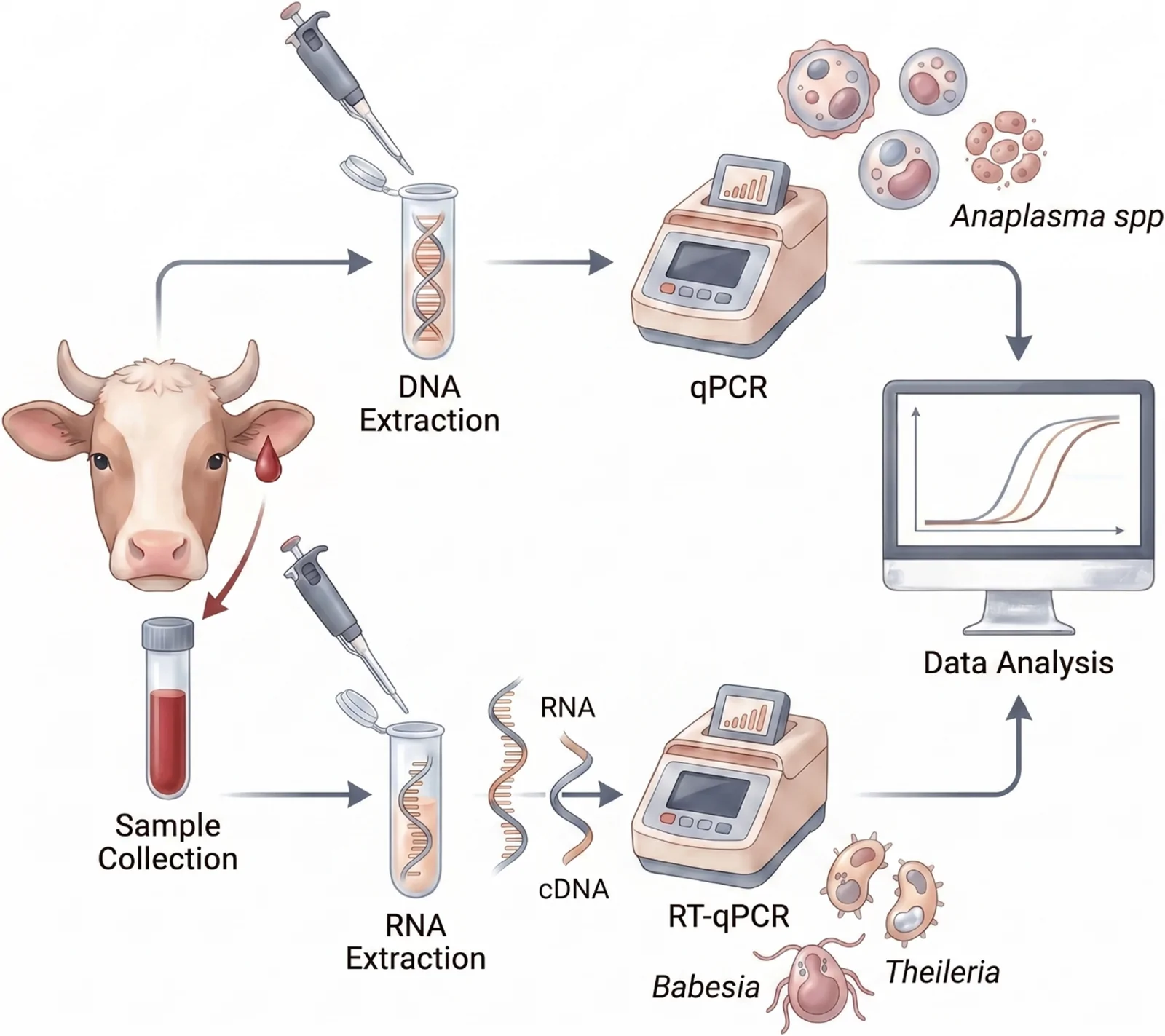
Schematic workflow illustrating the molecular detection of *Anaplasma* spp. and screening for hemoparasite co-infections in cattle using qPCR and RT-qPCR assays

Genomic DNA and total RNA were isolated from 200 µL of whole blood samples using commercial Qiagen kits according to the manufacturer’s instructions. The overall experimental workflow is illustrated in Fig. 1.

Detection of *A. marginale*, *A. centrale*, and *A. phagocytophilum* was performed using a DNA-based real-time quantitative PCR (qPCR) assay targeting the 16 S rRNA gene. Species differentiation was achieved using species-specific TaqMan probes based on previously validated protocols (Reinbold et al. 2010) (Table 1). qPCR reactions were carried out in a final volume of 25 µL using a Rotor-Gene Q 5plex HRM system (Qiagen, Germany), containing 12.5 µL of 2× QuantiTect Probe PCR Master Mix, 2 µL of 25× primer–probe mix, 5 µL of DNA template, and 5.5 µL of nuclease-free water. Thermal cycling conditions consisted of an initial activation step at 95 °C for 15 min, followed by 40 cycles of 94 °C for 15 s and 60 °C for 60 s. Samples with a quantification cycle (Cq) value < 38 were considered positive (Tables 2 and 3).

**Table 1:** TaqMan probes used in the qPCR assays

TaqMan probe	5’−3’ sequence	Length (nt)	Tm (°C)
*A. marginale* probe	5′-/56-FAM/CGCAGCTTGCTGCGTGTATGGT/3BHQ_1/−3′	22	62.8
*A. centrale*	5′-/5TexRd—*ATCATCATTCTTCCCCTTTACCTCGT_*BHQ2/−3’	26	60.8
*A. phagocytophilum* probe	5′-/5TexRd-XN/TTGCTATAAAGAATAATTAGTGGCAGACG/3BHQ_2/−3′	29	55.4

**Table 2:** *Anaplasma* agents detected in blood samples of infected cattle

**Sample No**	**Target result**	**Ct value**	**Pathogen type**
1	Positive	23	*A. marginale*
2	Positive	22	*A. marginale*
3	Positive	22	*A. marginale*
4	Positive	27	*A. marginale*
5	Positive	22	*A. centrale*
6	Positive	25	*A. centrale*
7	Positive	25	*A. centrale*
8	Positive	23	*A. centrale*
9	Positive	22/21	*A. marginale/A. centrale*
10	Positive	22/23	*A. marginale/A. centrale*
11	Positive	22/21	*A. marginale/A. centrale*
12	Positive	22/23	*A. marginale/A. centrale*

**Table 3:** Results of qPCR assay regression equations for the quantification of the 16 S rRNA gene

Assay	Regression equation	Correlation coefficient (*R*^2^)	Efficiency (%)
*A. marginale*	*y* = −3.4124*x* + 42.28	0.9947	96.5
*A. centrale*	*y* = −3.4124*x* + 42.28	0.9912	97.83

To investigate potential co-infections, *Babesia bigemina*,* B. bovis*, and *Theileria annulata* (*T. annulata*) were analyzed using RNA-based reverse transcription quantitative PCR (RT-qPCR). Total RNA was extracted from 200 µL of whole blood using the QIAamp RNA Blood Mini Kit (Qiagen, Cat. No: 52304). RNA concentration and purity were assessed using a NanoDrop spectrophotometer, and integrity was verified by agarose gel electrophoresis. To eliminate genomic DNA contamination, RNA samples were treated with DNase. Complementary DNA (cDNA) was synthesized using the QuantiTect Reverse Transcription Kit (Qiagen, Cat. No: 330411) according to the manufacturer’s instructions. RT-qPCR assays were performed using species-specific primers as previously described (Ros-García et al. 2012; Zhang et al. 2016). Reaction conditions and cycling parameters are provided in the supplementary data.

## Haematological analysis

A haematology analyser (Abacus Junior Vet5, Hungary) was used to determine white blood cell count (WBC), lymphocyte count, monocyte count, neutrophil count, eosinophil count, red blood cell count (RBC), haemoglobin concentration (HGB), haematocrit (HCT), mean corpuscular volume (MCV), mean corpuscular haemoglobin (MCH), mean corpuscular haemoglobin concentration (MCHC), red cell distribution width (RDW) and platelet count (PLT).

## Biochemical analysis

H-FABP and NT-proBNP serum concentrations were measured according to the manufacturer’s instructions using validated commercial bovine-specific enzyme-linked immunosorbent assay (ELISA) kits (Sunred Biological Technology, Shanghai, China). With a minimum detectable concentration (MDC) of 0.08 ng/mL, the intra-assay and inter-assay coefficients of variation (CV) for H-FABP were reported to be < 10% and < 12%, respectively. The intra-assay and inter-assay CVs for NT-proBNP were also < 10% and 12%, respectively, with an MDC of 0.01 ng/mL. According to the manufacturer’s validation data, both assays demonstrated appropriate analytical performance in bovine serum, including defined sensitivity (H-FABP: 0.074 ng/mL; NT-proBNP: 0.008447 ng/mL), broad assay ranges (H-FABP: 0.08–20 ng/mL; NT-proBNP: 0.01–3 ng/L), and acceptable intra- and inter-assay precision based on repeated measurements across multiple plates. In addition, previous studies have successfully applied these kits in cattle, supporting their suitability for bovine cardiac biomarker analysis (Yildiz et al. 2019; Değirmençay 2023). The concentrations of cTnI in the serum were measured using a commercial immunoassay system based on a one-step sandwich method (Unicel Beckman Coulter Access II, USA). The analytical measurement range of the assay was 0.01–100 ng/mL, with a limit of detection of 0.01 ng/mL. Serum CK, CK-MB, LDH, AST, alanine aminotransferase (ALT), alkaline phosphatase (ALP), and gamma-glutamyl transferase (GGT) activities and the concentrations of total bilirubin (TBIL), direct bilirubin (DBIL), total protein (TP), albumin (ALB), glucose, blood urea nitrogen (BUN), and creatinine (Cr) were determined using a biochemistry autoanalyzer (Beckman Coulter, AU5800, USA) employing commercial enzyme kits.

### Statistical analysis

For statistical analysis, the SPSS software program (Version 27.0, SPSS Inc., Chicago, IL, USA) was used. The Shapiro-Wilk test was applied to assess the normality of data distribution between the two main groups (anaplasmosis and healthy control). The independent-samples *t*-test was used to compare parametric variables, while the Mann-Whitney *U* test was applied for nonparametric variables. The Spearman correlation test was used to evaluate associations among parameters, as several variables did not meet the assumption of normal distribution. Correlation coefficients were interpreted as weak (0.10–0.39), moderate (0.40–0.69), strong (0.70–0.89), or very strong (0.90–1.00) (Mukaka 2012).

To further investigate the independent predictors of myocardial injury biomarkers, multiple linear regression analyses were performed using heart rate (HR) and haematocrit (HCT) as explanatory variables and H-FABP and NT-proBNP as dependent variables. Model fit was evaluated using the coefficient of determination (R²), and standardized beta coefficients (β) were used to determine the relative contribution of each predictor.

A histopathology- or imaging-based gold standard for myocardial injury was not available in this study. Therefore, cTnI was used as a surrogate reference biomarker for receiver operating characteristic (ROC) analysis. Animals were classified according to cTnI concentrations exceeding 0.064 ng/mL, which was identified as the optimal diagnostic cut-off value based on ROC curve analysis. ROC curves for H-FABP and NT-proBNP were constructed using cTnI-defined myocardial injury status as the reference standard. The diagnostic cut-off values of H-FABP and NT-proBNP indicating myocardial injury were determined using ROC curve analysis. For each variable, the sensitivity, specificity, and area under the curve (AUC) were calculated. The AUC categorizations denote the predictive capacity as follows: bad (0.50–0.60), poor (0.60–0.70), fair (0.70–0.80), good (0.80–0.90), or excellent (0.9–1) (Swets 1988). The formula [PPV = TP/(TP + FP) × 100] was used to calculate the positive predictive value (PPV) for cardiac injury, using the true positive (TP) and false positive (FP) values for each variable.

To further explore group differences, animals in the anaplasmosis group were subdivided into three subgroups according to qPCR results: Mixed infection, *A. marginale*, and *A. centrale*. Together with the healthy controls, this yielded four comparison groups in total. For multiple group comparisons, one-way analysis of variance (ANOVA) followed by Duncan’s multiple range test was performed for parametric data, while the Kruskal–Wallis H test was applied for nonparametric data. Parametric data were expressed as mean ± standard deviation (SD), and nonparametric data as median (min–max). A *P* value of less than 0.05 was considered statistically significant for all comparisons. Given the small sample size in subgroup analyses (*n* = 4 per group), these comparisons were considered exploratory, should be interpreted with caution, and are not intended for definitive statistical inference. A formal sample size calculation was not performed due to the exploratory nature of the study and limited availability of clinically affected animals.

## Results

### Clinical findings

The predominant clinical signs in the cattle with anaplasmosis were pyrexia (RT: 39.4 ± 0.78 °C), tachycardia (118 ± 17.4 beats/min), increased respiratory rate (32.3 ± 6.02 breaths/min), pale or icteric mucous membranes, weakness, and anorexia. Infected cattle exhibited significantly higher RT, HR, and RR compared to the healthy control group (*P* < 0.001) (Table 4). No concurrent diseases were detected in the infected animals.

**Table 4:** Comparison of haematological, biochemical and some clinical findings of cattle in the infected and control groups

Parameters	Unit	Healthy (*n* = 12)	95% Confidence Interval	Anaplasmosis (*n* = 12)	95% Confidence Interval	*P* value
RT	°C	38.4 ± 0.25	38.3–38.6	39.4 ± 0.78	39.0–39.9	**< 0.001**
RR	breaths/min	21.7 ± 3.17	20–23.4	32.3 ± 6.02	29.1–35.6	**< 0.001**
HR	beats/min	67.3 ± 6.79	63.5–71.3	118 ± 17.4	108–127	**< 0.001**
WBC	x10^3^/µL	6.87 (4.26–8.04)	6.20–7.24	10.8 (6.53–29.4)	8.18–13.3	**< 0.001**
Lymphocyte count	x10^3^/µL	3.95 (2.68–4.79)	3.54–4.29	6.65 (3.79–14.6)	4.99–7.78	**0.002**
Monocyte count	x10^3^/µL	0.26 ± 0.11	0.20–0.33	0.41 ± 0.19	0.29–0.52	**0.035**
Neutrophil count	x10^3^/µL	2.28 ± 0.89)	1.80–2.77	4.76 ± 3.88	2.88–7.23	0.052
Eosinophil count	x10^3^/µL	0.09 (0.03–0.25)	0.05–0.14	0.08 (0.04–0.39)	0.05–0.11	0.642
RBC	x10^6^/µL	5.89 ± 0.45	5.63–6.15	4.63 ± 2.17	3.43–5.87	0.070
HGB	g/dL	8.48 ± 0.76	8.08–8.93	6.34 ± 2.53	4.98–7.79	**0.015**
HCT	%	25.4 ± 2.65	24.1–27.0	17.2 ± 7.04	13.5–21.3	**0.002**
MCV	fL	44.5 (37–48)	39.5–45.5	37 (31–66)	35–39	**0.015**
MCH	pg	14.4 (12.8–15.4)	13.5–15.1	13.4 (12.1–25.4)	12.5–15.4	0.236
MCHc	g/dL	33.5 ± 1.19	32.9–34.2	37.2 ± 3.56	35.3–39.4	**0.005**
RDW	%	23.2 ± 2.20	22–24.6	27.3 ± 2.74	25.9–29.1	**< 0.001**
PLT	x10^3^/µL	243 (38–981)	171–280	357 (97–613)	266–405	0.094
H-FABP	ng/mL	0.43 ± 0.06	0.39–0.46	0.64 ± 0.05	0.61–0.66	**< 0.001**
NT-proBNP	ng/mL	0.21 ± 0.06	0.18–0.24	0.36 ± 0.09	0.31–0.41	**< 0.001**
cTnI	ng/mL	0.06 (0.04–0.07)	0.04–0.06	0.11 (0.03–0.34)	0.07–0.14	**0.003**
CK-MB	U/L	119 ± 21.5	108–133	160 ± 58	130–195	**0.041**
CK	U/L	187 ± 73.2	149–229	1193 ± 980	645–1747	**0.005**
LDH	U/L	937 ± 193	835–1054	1874 ± 589	1499–2181	**< 0.001**
AST	U/L	55.4 ± 6.78	52–60	156 ± 75	115–197	**< 0.001**
ALT	U/L	31.7 ± 10	26–37	30.7 ± 9.5	25.7–35.8	0.808
ALP	U/L	49 (17–268)	25–97	94.5 (45–148)	67.5–113	0.069
GGT	U/L	11.9 ± 4.60	9.22–14.7	23.4 ± 16.3	15.3–33.5	0.035
TBIL	mg/dL	0.02 (0.01–0.06)	0.02–0.05	0.35 (0.01–3.30)	0.16–1.06	**< 0.001**
DBIL	mg/dL	0.02 (0.01–0.03)	0.02–0.03	0.11 (0–0.67)	0.03–0.27	**0.031**
TP	g/dL	6.20 ± 1.19	5.55–6.86	6.13 ± 0.78	5.69–6.56	0.871
ALB	g/dL	2.57 ± 0.48	2.31–2.84	2.32 ± 0.34	2.11–2.49	0.148
BUN	mg/dL	10.9 (9.84–15.8)	10.1–13.5	31.7 (12.4–76.2)	23.8–46.1	**< 0.001**
Cr	mg/dL	0.91 (0.79–1.14)	0.87–1.02	0.93 (0.22–4.35)	0.65–1.04	0.862
Glucose	mg/dL	61.1 ± 12.1	54.4–67.4	69.4 ± 13.4	62.2–76.9	0.125

RT: Rectal temperature; HR: Heart rate (per min); RR: Respiratory rate (per min); WBC: white blood cell; RBC: red blood cell; HGB: hemoglobin; HCT: hematocrit; MCV: mean erythrocyte volume; MCH: mean erythrocyte hemoglobin; MCHC: mean erythrocyte hemoglobin concentration; RDW: erythrocyte distribution width; PLT: platelet; H-FABP: heart type fatty acid binding protein; NT-proBNP: N-terminal pro-peptide natriuretic type B; cTnI: cardiac troponin I; CK-MB; creatine kinase myocardial band; CK; creatine kinase; LDH: lactate dehydrogenase; AST: aspartate aminotransferase; ALT: alanine aminotransferase; ALP: alkaline phosphatase; GGT: gamma glutamyl transferase; TBIL: total bilirubin; DBIL: direct bilirubin; TP: total protein; ALB: albumin; BUN: blood urea nitrogen; Cr: creatinine; Parametric data are expressed as mean ± SD, and nonparametric data are expressed as median (min–max)

### Microscopic findings

**Fig. 2:**
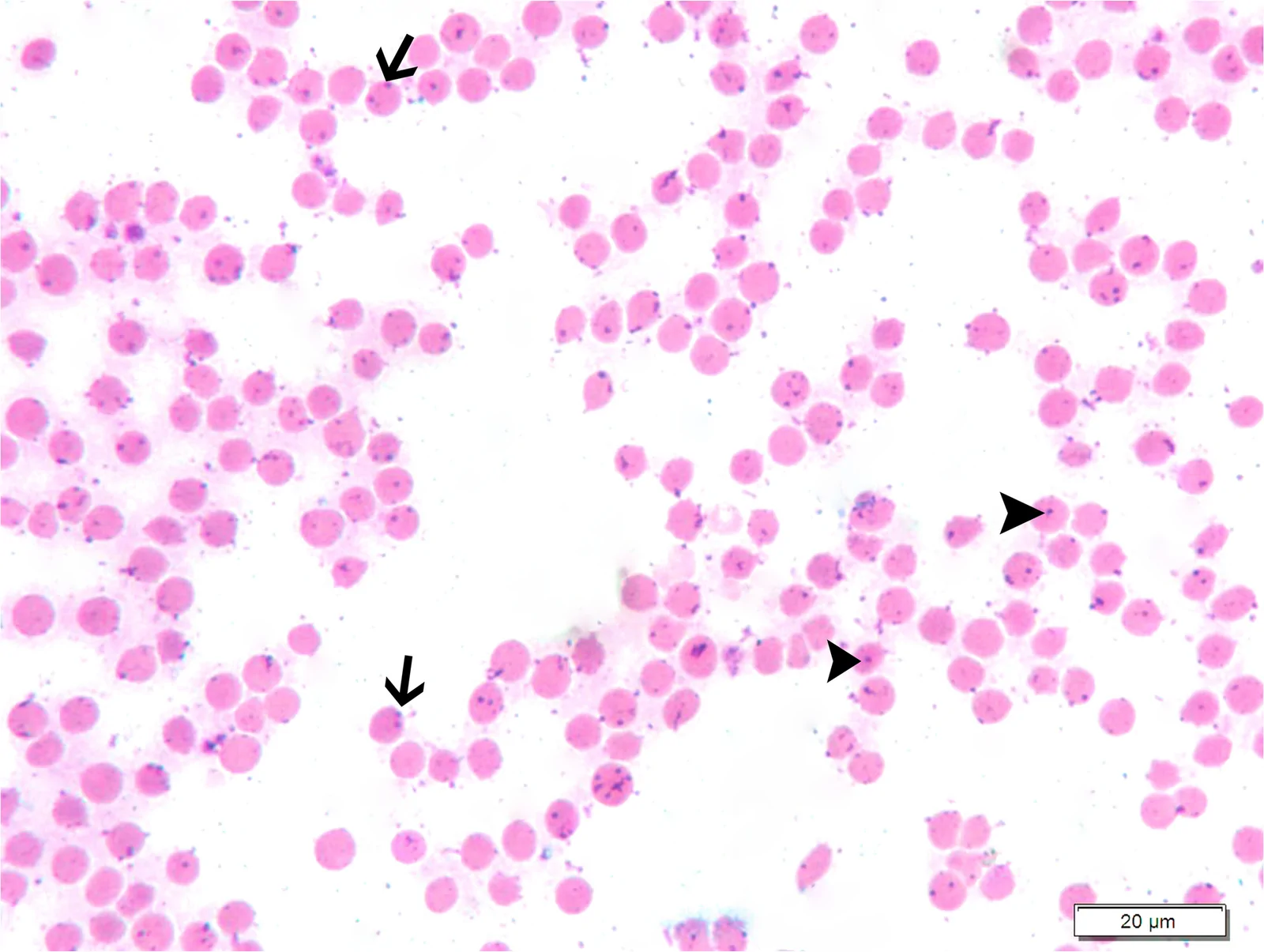
Representative Wright–Giemsa-stained blood smears images showing intraerythrocytic inclusions morphologically consistent with *Anaplasma* spp. in cattle. Arrows indicate qPCR-confirmed *A. marginale* cases, whereas arrowheads indicate qPCR-confirmed *A. centrale* cases (×1000 oil immersion; scale bar = 20 μm)

Microscopic examination of Wright–Giemsa-stained blood smears revealed intraerythrocytic inclusions morphologically consistent with *Anaplasma* spp. in all infected animals (Fig. 2). Microscopy was used as a supportive diagnostic tool for visualization of parasitized erythrocytes and estimation of parasitaemia. Parasitaemia ranged between approximately 20% and 80% of erythrocytes in microscopic fields. No *Babesia* spp. or *Theileria* spp. were detected during the microscopic examination. Definitive species identification and determination of co-infections were based on qPCR and RT-qPCR analyses.

### Molecular detection findings

qPCR analysis was used for definitive identification of *A. marginale*, *A. centrale*, and *A. phagocytophilum* in bovine peripheral blood samples. Four of the 12 samples were classified as *A. marginale* alone, 4 as *A. centrale* alone, and 4 as mixed infections of *A. marginale* and *A. centrale* (Table 2). The qPCR assay regression equations for the 16 S rRNA of *A. marginale* and *(A) centrale* demonstrated efficiencies of 96.50% and 97.83% (R^2^ = 0.99), respectively (Table 3). RT-qPCR analysis did not detect the presence of *(B) bigemina*, *B. bovis*, or *T. annulata* in any sample (see Supplementary data, Table S1).

### Haematological findings

Cattle with anaplasmosis had significantly higher median WBC (*P* < 0.001) and lymphocyte counts (*P* = 0.002) as well as mean monocyte counts (*P* = 0.035), alongside significantly lower mean HGB concentrations (*P* = 0.015) and HCT values (*P* = 0.002), and median MCV levels (*P* = 0.015) compared to the healthy group. As expected, RDW values were significantly higher (*P* < 0.001) (Table 2). Upon categorizing the infected animals into three groups—Mixed infection (*n* = 4), *A. marginale* (*n* = 4), and *A. centrale* (*n* = 4)—based on the identified agent, WBC and lymphocyte counts were higher in the mixed infection and *A. marginale* groups compared to healthy controls, whereas values in the *A. centrale* group were closer to those of the control group. In parallel, the Mixed infection group exhibited the lowest erythrocyte parameters (RBC, HGB, and HCT), followed by the *A. marginale* and *A. centrale* groups (Supplementary Table S2).

### Biochemical findings

The mean concentrations of H-FABP and NT-proBNP in the infected group were significantly higher than those in the control group (*P* < 0.001). Median concentrations of cTnI (*P* = 0.003) and BUN (*P* < 0.001), as well as mean CK-MB (*P* = 0.041), CK (*P* = 0.005), LDH, and AST (*P* < 0.001), were also significantly elevated in the infected group. Consistent with the jaundice observed in cattle with anaplasmosis, TBIL (*P* < 0.001) and DBIL (*P* = 0.031) concentrations were significantly higher in the infected group. However, ALT, TP, ALB, ALP, GGT, Cr, and glucose concentrations did not significantly differ between the infected and control groups (Table 2). The Mixed infection, *A. marginale*, and *A. centrale* groups showed the highest serum concentrations of H-FABP, NT-proBNP, and TBIL, respectively. In addition, CK, LDH, and AST activities tended to be higher in the *A. marginale* and Mixed infection groups compared with the control group (Supplementary Table S2). These patterns were particularly evident in the Mixed infection group.

### Correlation analysis

**Fig. 3:**
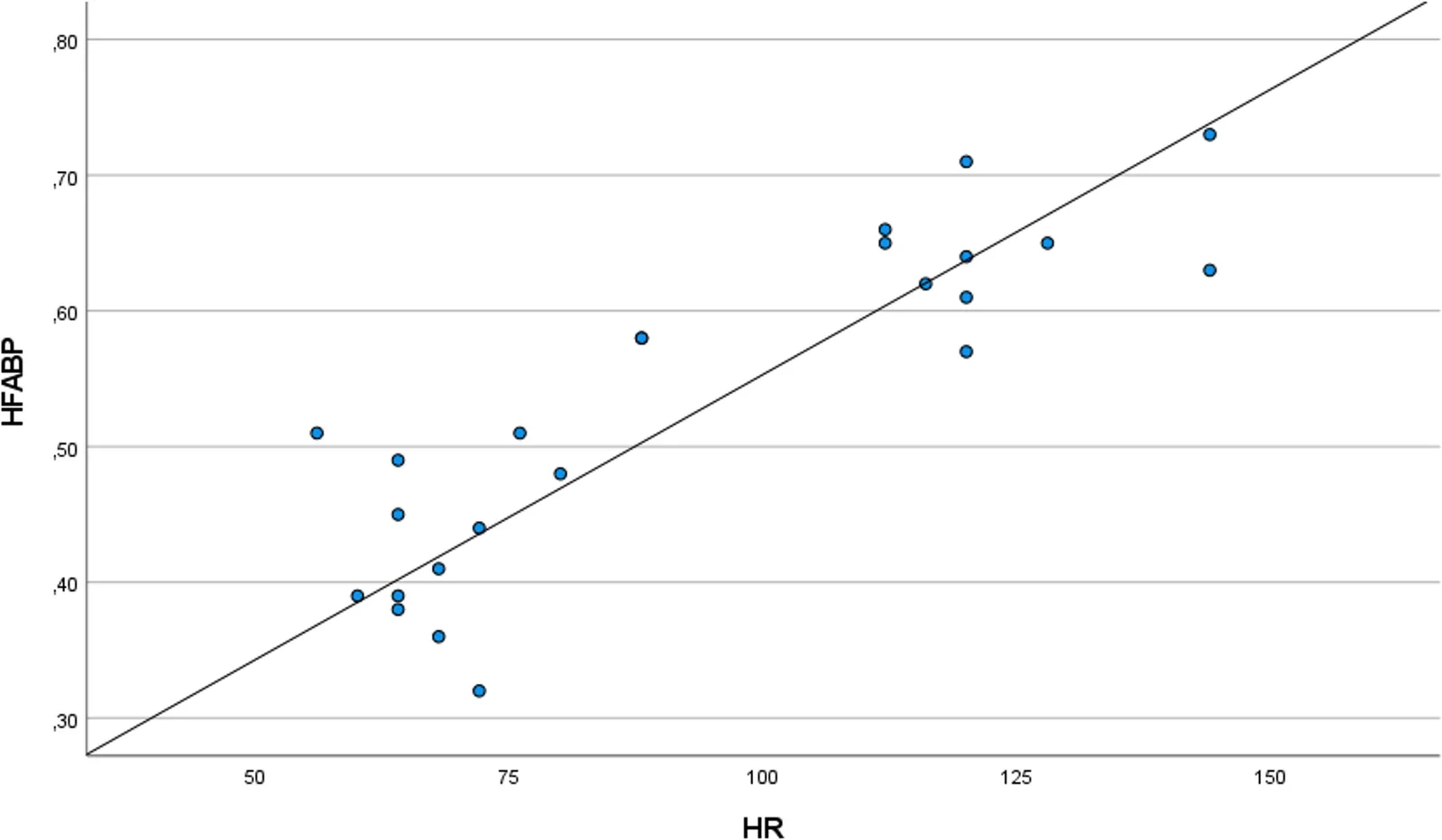
Scatter plot showing the relationship between heart rate (HR) and H-FABP concentrations in cattle

**Fig. 4:**
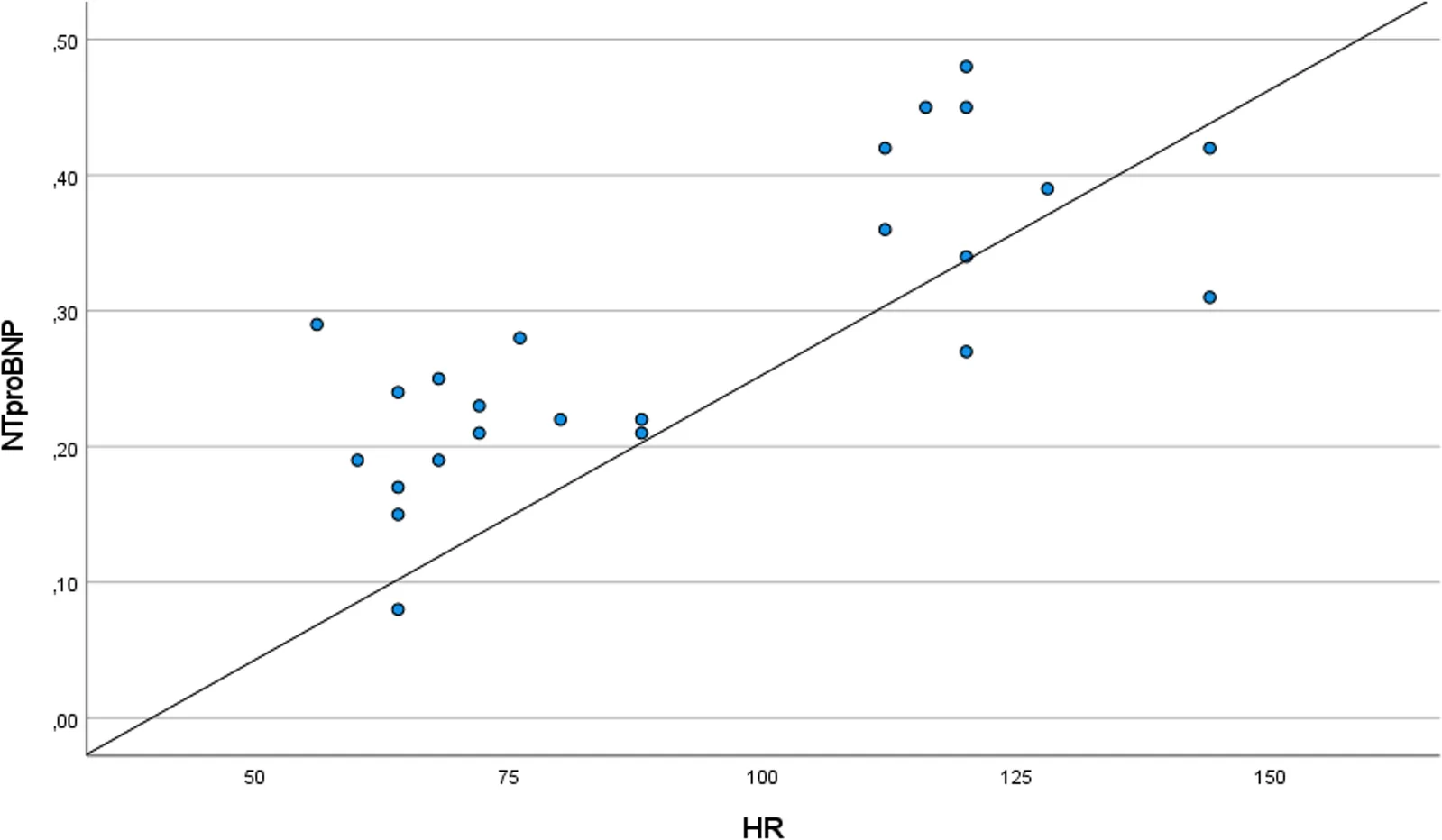
Scatter plot showing the relationship between heart rate (HR) and NT-proBNP concentrations in cattle

Correlation analysis demonstrated significant associations between hematologic indicators of anaemia, heart rate, and cardiac biomarkers. HCT showed moderate negative correlations with H-FABP (*r* = − 0.413, *P* = 0.045) and NT-proBNP (*r* = − 0.617, *P* = 0.001). Heart rate showed strong positive correlations with H-FABP (*r* = 0.803, *P* < 0.001) and NT-proBNP (*r* = 0.732, *P* < 0.001), and a moderate positive correlation with cTnI (*r* = 0.537, *P* = 0.007). In addition, H-FABP showed a strong positive correlation with NT-proBNP (*r* = 0.740, *P* < 0.001) and a moderate positive correlation with cTnI (*r* = 0.581, *P* = 0.003) (Table 5). Scatter plot analyses further illustrated the strong positive relationships between heart rate and the cardiac biomarkers H-FABP and NT-proBNP (Figs. 3 and 4).

**Table 5:** Correlation results between hematologic parameters and cardiac biomarkers of cattle in the infected and control groups (Spearman Correlation)

Parameters	HGB	HCT	HR	H-FABP	NT-pro BNP	cTnI
HGB	1.000	0.980**	−0.505*	−0.354	−0.571**	−0.307
HCT		1.000	−0.561**	−0.413*	−0.617**	−0.390
HR			1.000	0.803**	0.732**	0.537**
H-FABP				1.000	0.740**	0.581**
NT-proBNP					1.000	0.641**
cTnI						1.000

*HGB* hemoglobin, *HCT *haematocrit, *HR *heart rate, *H-FABP *heart type fatty acid binding protein, *NT-proBNP *N-terminal pro-peptide natriuretic type B, *cTnI *cardiac troponin I

*P < 0.05, **P < 0.01

### Multiple regression analysis

Multiple linear regression analysis was performed to determine whether heart rate and haematocrit independently predicted myocardial injury biomarkers. For NT-proBNP, the regression model was significant (R² = 0.667, adjusted R² = 0.635, *P* < 0.001). Heart rate was identified as an independent predictor of NT-proBNP concentrations (β = 0.549, *P* = 0.004), whereas haematocrit was not significantly associated with NT-proBNP concentrations (β = −0.338, *P* = 0.062).

Similarly, regression analysis for H-FABP revealed a significant model (R² = 0.765, adjusted R² = 0.743, *P* < 0.001). Heart rate remained a strong independent predictor of H-FABP concentrations (β = 0.974, *P* < 0.001), while haematocrit was not independently associated with H-FABP levels (β = 0.159, *P* = 0.283).

These findings indicate that myocardial injury biomarkers are strongly associated with heart rate and inversely associated with hematologic indicators of anaemia.

### ROC analysis

**Fig. 5:**
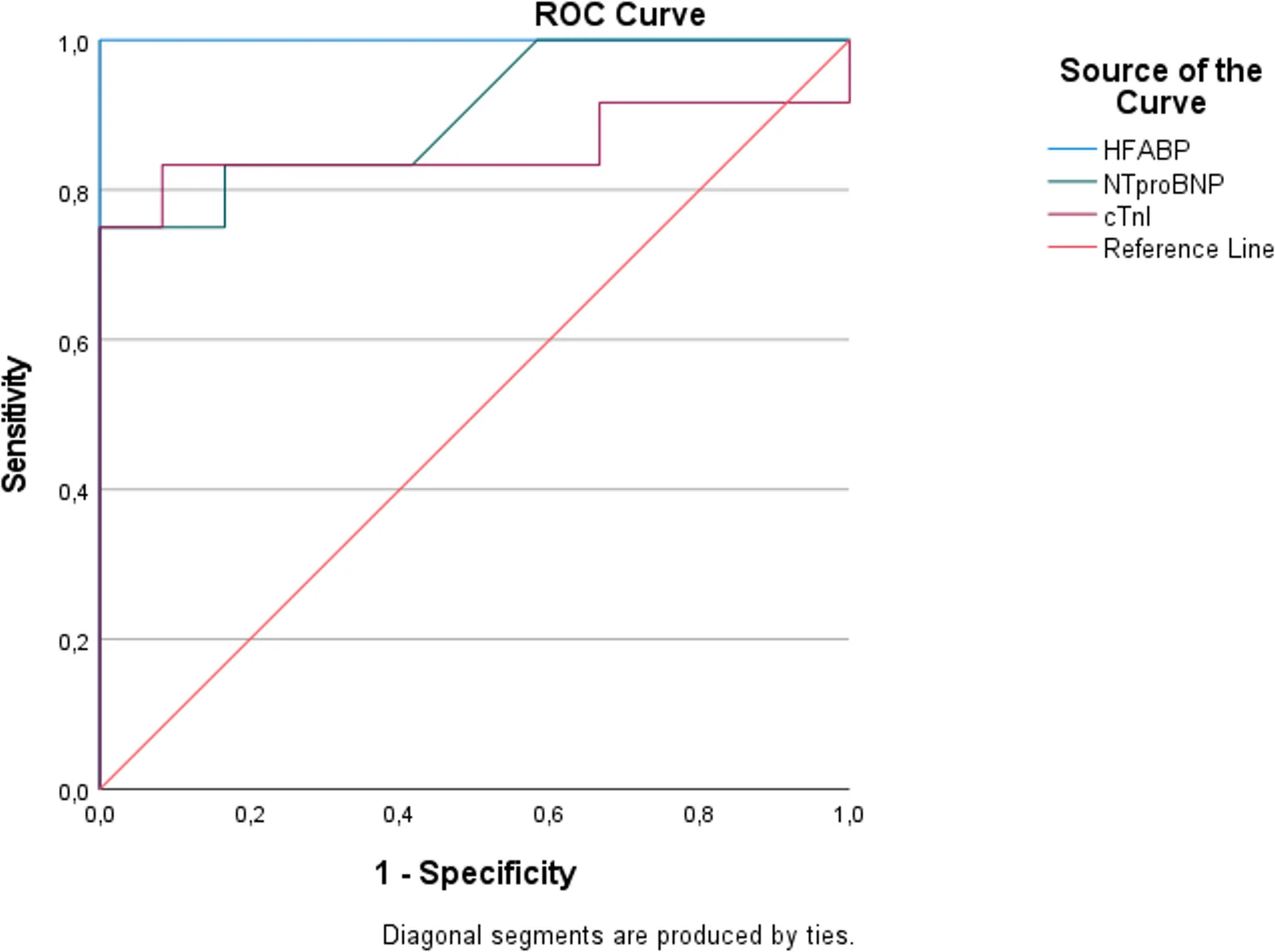
Receiver operating characteristic (ROC) curves of H-FABP, NT-proBNP, and cTnI for detecting myocardial injury in cattle with anaplasmosis. The area under the curve (AUC) values and corresponding 95% confidence intervals (CI) are presented in Table 5

Table 6; Fig. 5 present the ROC analysis results of cardiac biomarkers. The ROC analysis for H-FABP estimated an AUC of 1.000 (*P* < 0.001), with a diagnostic cut-off value of 0.51 ng/mL, indicating cardiac damage with 100% sensitivity and 100% specificity (100% PPV). NT-proBNP and cTnI exhibited AUC values of 0.903 and 0.854, respectively. The 95% confidence intervals (CI) for AUC values are presented in Table 5. NT-proBNP levels above 0.29 ng/mL (100% PPV) and cTnI levels above 0.064 ng/mL (90.9% PPV) were indicative of cardiac damage, demonstrating 75% sensitivity and 100% specificity for NT-proBNP, and 83.3% sensitivity and 91.7% specificity for cTnI. Overall, H-FABP showed the best diagnostic performance among the evaluated biomarkers, followed by NT-proBNP, whereas cTnI demonstrated comparatively lower diagnostic accuracy.

**Table 6:** ROC analysis results of H-FABP, NT-proBNP, and cTnI

Parameters	H-FABP (ng/mL)	NT-proBNP (ng/mL)	cTnI (ng/mL)
Area	1.000	0.903	0.854
Cut-off	> 0.51	> 0.29	> 0.064
Sensitivity (%)	100	75	83.3
Specificity (%)	100	100	91.7
SE	0.000	0.063	0.093
P value	0.000	0.001	0.003
PPV (%)	100	100	90.9
95% CI Lower Bound	1.000	0.779	0.672
95% CI Upper Bound	1.000	1.000	1.000

*H-FABP *heart type fatty acid binding protein, *NT-proBNP *N-terminal pro-peptide natriuretic type B, *cTnI *cardiac troponin I, *SE *standard error, *PPV *positive predictive value, *CI *Confidence Interval

## Discussion

The present study demonstrated that bovine anaplasmosis is associated with significant myocardial injury, as evidenced by increased concentrations of H-FABP, NT-proBNP, cTnI, CK-MB, CK, AST, and LDH. These findings support our initial hypothesis that severe anaemia and secondary tissue hypoxia in anaplasmosis may lead to myocardial damage. Previous pathological investigations have described myocardial degeneration and coagulative necrosis in cattle infected with *A. marginale* (Jaswal et al. 2015), suggesting that the cardiovascular system may be affected during the course of the disease. In the present study, cardiac biomarker concentrations increased in parallel with the severity of anaemia, and the highest levels were observed in the mixed infection group. In addition, infected cattle exhibited significantly higher heart rates than healthy controls, and heart rate showed strong positive correlations with several cardiac injury markers. These findings suggest that tachycardia represents a compensatory response to reduced oxygen-carrying capacity and tissue hypoxia. Sustained tachycardia may further aggravate myocardial oxygen imbalance by shortening diastolic perfusion time while simultaneously increasing myocardial oxygen demand (Zellweger et al. 2003; Miranda et al. 2006). Therefore, myocardial injury observed in anaplasmosis is most likely a consequence of severe anaemia-induced hypoxia and increased cardiac workload rather than a direct cytopathic effect of the pathogen. This interpretation is further supported by the observed correlations between cardiac biomarkers and haemolysis-related biochemical markers such as LDH and AST. Myocardial injury biomarkers were strongly associated with anaemia severity and tachycardia, suggesting a potential pathophysiological link between hypoxia and cardiac involvement rather than a direct causal relationship.

The haematological findings of this study clearly demonstrate that bovine anaplasmosis is characterized by haemolytic anaemia accompanied by compensatory hematologic responses. Several previous studies have reported significantly decreased RBC, HGB, and HCT levels in cattle infected with *A. marginale* (De et al. 2012; Nazifi et al. 2012; Esmaeilnejad et al. 2018). Consistent with these reports, the infected animals in the present study exhibited marked reductions in HGB, HCT, and MCV values, accompanied by increased RDW values. These alterations indicate anisocytosis and enhanced erythropoietic activity, which typically occur as a compensatory response to haemolysis; however, this was not specifically quantified by microscopic grading in the present study. The destruction of infected erythrocytes by the reticuloendothelial system and antibody-mediated erythrophagocytosis have been proposed as the principal mechanisms responsible for anaemia in anaplasmosis (De et al. 2012). Furthermore, the elevated concentrations of TBIL observed in the infected cattle are consistent with haemolytic processes and support the occurrence of erythrocyte destruction. Together, these haematological and biochemical alterations strongly indicate that haemolytic anaemia represents a central pathophysiological component of bovine anaplasmosis.

In addition to erythrocyte alterations, infected animals showed leucocytosis accompanied by lymphocytosis and monocytosis. These changes are consistent with activation of the immune response during persistent *Anaplasma* infection (Han et al. 2010). Monocytosis may also reflect increased phagocytic activity associated with the removal of damaged erythrocytes and cellular debris in the spleen and liver (Borges and Sesti-Costa 2022). Similar haematological alterations have previously been reported in cattle with naturally occurring anaplasmosis (Nazifi et al. 2012; Asif et al. 2022). Therefore, haematological indices such as RDW and leukocyte differential counts may provide useful auxiliary indicators for evaluating disease severity and inflammatory status in cattle affected by this infection (Roland et al. 2014; Miglio et al. 2023).

Although cardiac involvement in hemoparasitic diseases has been documented in infections such as theileriosis and babesiosis (Fartashvand et al. 2013; Bartnicki et al. 2017; Ahmadpour et al. 2020; Değirmençay et al. 2021; Rasoulzadeh et al. 2021), relatively limited information is available regarding myocardial injury associated specifically with bovine anaplasmosis. Previous investigations have reported increased cTnI concentrations in dairy cows naturally infected with anaplasmosis, indicating the presence of myocardial injury in affected animals (Jayalakshmi et al. 2020). Furthermore, a recent study evaluating cardiac biomarkers in cattle naturally infected with anaplasmosis, theileriosis, and babesiosis highlighted the potential diagnostic value of cardiac biomarkers for detecting myocardial damage in hemoparasitic infections (Yogeshpriya et al. 2022). In addition, histopathological investigations have demonstrated myocardial degeneration and necrosis in cattle infected with *A. marginale* (Jaswal et al. 2015; Das et al. 2021). In this context, the present study provides further biochemical evidence supporting the presence of myocardial injury during clinical bovine anaplasmosis.

In the present study, cardiac biomarker concentrations increased in parallel with the severity of anaemia. The lowest erythrocyte parameters and the highest H-FABP, NT-proBNP, and cTnI levels were detected in the Mixed infection, A. marginale, and A. centrale groups, respectively. These findings suggest that myocardial injury may intensify as anaemia becomes more severe. The results are consistent with previous studies demonstrating that severe anaemia and tissue hypoxia may trigger myocardial ischemia and necrosis (Fartashvand et al. 2013). Among the evaluated biomarkers, H-FABP showed the best diagnostic performance in ROC analysis, with an AUC value of 1.000 and 100% sensitivity and specificity. H-FABP is a small cytoplasmic protein abundantly present in cardiomyocytes and is rapidly released into the circulation following myocardial injury (Liebetrau et al. 2014). Its rapid release kinetics makes it particularly useful for detecting early myocardial injury. Experimental studies in non-bovine species and experimental cellular models have demonstrated a potential anti-apoptotic role of H-FABP in hypoxia-reoxygenation injury and its modulation by anti-tachycardic therapies (Zhang et al. 2017; Rezar et al. 2020). Therefore, the superior diagnostic performance observed in this study may be attributed to the rapid release of H-FABP following even minor myocardial damage compared with structural biomarkers such as cardiac troponins (Iida et al. 2007). Importantly, this study provides a clinically relevant biomarker-based framework for detecting myocardial injury in bovine anaplasmosis under field conditions, where advanced diagnostic tools such as echocardiography are often unavailable.

NT-proBNP also demonstrated high diagnostic accuracy for detecting cardiac injury, ranking as the second-best biomarker in ROC analysis. NT-proBNP is released from ventricular myocardium in response to increased myocardial wall tension, pressure overload, and hypoxic stress (Hall 2004; Potter et al. 2009). In the present study, NT-proBNP concentrations showed strong positive correlations with heart rate and moderate negative correlations with erythrocyte indices, suggesting that reduced oxygen-carrying capacity may increase myocardial wall stress through hypoxia-induced tachycardia. Similar increases in NT-proBNP concentrations have been reported in dogs with babesiosis and were associated with disease severity and cardiovascular stress (Lobetti et al. 2012). These findings support the concept that natriuretic peptides may serve as useful biomarkers for detecting cardiac stress associated with systemic infectious diseases.

It should also be noted that low concentrations of cardiac biomarkers such as H-FABP, NT-proBNP, and cTnI may be detectable in clinically healthy animals, reflecting normal cardiomyocyte turnover and basal physiological processes. However, the significantly higher concentrations observed in infected cattle in the present study indicate that these biomarkers are sensitive indicators of myocardial stress and injury rather than being entirely disease-specific. Therefore, their diagnostic value lies in the magnitude of increase relative to baseline levels rather than their mere presence in circulation.

Although cTnI ranked third in ROC analysis, infected cattle exhibited approximately twice the cTnI concentrations observed in healthy animals, confirming its diagnostic relevance for myocardial injury. The comparatively lower diagnostic performance of cTnI in ROC analysis may be explained by its release kinetics, as cTnI primarily reflects structural myocardial injury, whereas H-FABP is rapidly released even in early and mild ischemic conditions. In this context, H-FABP may be more sensitive for detecting early or subclinical myocardial injury, while cTnI remains a highly specific marker of established cardiomyocyte damage. Furthermore, it should be considered that cTnI was used as the reference standard in ROC analysis, which may inherently limit its apparent diagnostic performance when compared with alternative biomarkers. This approach may introduce a degree of circularity bias, as cTnI was used both as a reference standard and as a comparator biomarker. Therefore, the reported sensitivity and specificity values should be interpreted as relative diagnostic performance against the selected reference biomarker rather than absolute measures of diagnostic accuracy. Therefore, the diagnostic performance of alternative biomarkers should be interpreted in this context. Future studies incorporating independent clinical or imaging-based reference standards would further strengthen these findings. Therefore, these findings suggest that H-FABP and NT-proBNP may serve as complementary biomarkers that improve early detection of myocardial involvement in bovine anaplasmosis. As the most cardiac-specific biomarker available, cTnI is highly sensitive for detecting myocardial injury and is more specific than CK, CK-MB, LDH, or AST (Maynard et al. 2000; Gupta et al. 2018). In cattle, cTnI is considered the most cardiac-specific and extensively validated biochemical indicator of myocardial injury (Varga et al. 2009). The high sequence homology of cardiac troponin I between humans and cattle (> 96%) supports the applicability of commercially available immunoassays for use in bovine species (O’Brien et al. 1997). Its elevation in cattle with anaplasmosis therefore indicates cardiomyocyte damage most likely secondary to anaemia-induced hypoxia and compensatory tachycardia. However, because the present study did not include animals with anaemia caused by non-infectious conditions such as iron deficiency or haemorrhage, a direct comparison between anaplasmosis-associated anaemia and other aetiologies could not be performed. It should also be considered that circulating cTnI concentrations may increase in animals with renal dysfunction due to reduced renal clearance (Apple et al. 2002). Nevertheless, serum creatinine concentrations did not differ significantly between infected and control animals in the present study, suggesting that the observed elevation of cTnI was more likely associated with myocardial injury rather than impaired renal elimination. Although serum creatinine concentrations did not differ significantly between groups, it should be noted that creatinine is an insensitive marker of early renal dysfunction, and subclinical renal effects on biomarker clearance cannot be completely excluded. Similar increases in cTnI concentrations have been reported in other hemoparasitic diseases such as babesiosis and theileriosis (Diana et al. 2007; Fartashvand et al. 2013; Orunç Kilinç et al. 2015; Razavi et al. 2015), where hypoxia-related myocardial injury represents an important component of disease pathophysiology.

Another notable finding of the present study was that the most severe hematologic impairment and highest cardiac biomarker concentrations were observed in the Mixed infection group. This observation suggests that coinfection with *A. marginale* and *A. centrale* may exacerbate disease severity. The higher virulence of *A. marginale* compared with *A. centrale* has been well documented (Potgieter and Stoltsz 2004; Kocan et al. 2010; Hove et al. 2018), and coinfections may intensify pathogenic effects through cumulative erythrocyte destruction and systemic inflammatory responses. In this context, systemic inflammation may contribute to myocardial injury through cytokine-mediated mechanisms, oxidative stress, and endothelial dysfunction, thereby amplifying cardiac damage beyond the effects of anaemia-induced hypoxia alone. Importantly, no *Babesia* spp., *Theileria* spp., or *A. phagocytophilum* were detected in the present study. This finding eliminates potential confounding effects of other hemoparasitic infections and strengthens the association between bovine anaplasmosis, and myocardial injury observed in this investigation.

This study has several limitations. First, blood gas analysis was not performed, which prevented direct confirmation of systemic hypoxia. Nevertheless, clinical findings such as tachycardia, tachypnoea, and severe anaemia strongly support the presence of tissue hypoxia. Second, the relatively small sample size, particularly within subgroup analyses, represents another limitation of the study. Although the number of animals was sufficient to detect significant overall differences between infected and control groups, subdivision of infected animals reduced statistical power. Subgroup comparisons should therefore be interpreted cautiously due to the limited sample size and were considered exploratory in nature, rather than definitive statistical inferences. These findings should be confirmed in larger, well-powered studies to validate subgroup-specific differences. Another limitation of the present study is that animals were enrolled over a relatively short time period, which may affect the generalizability of the findings. All animals received standard treatment for anaplasmosis following sample collection and diagnosis, including long-acting oxytetracycline (20 mg/kg, intramuscularly, administered twice at 72 h intervals), imidocarb dipropionate (3 mg/kg, intramuscularly, single dose), antipyretic therapy when required, and supportive fluid therapy according to clinical status. No mortality was observed during the study period, and post-mortem examination was therefore not performed. Future investigations including larger populations, echocardiographic evaluation, serial biomarker monitoring, and myocardial histopathology would provide a more comprehensive understanding of cardiac involvement in bovine anaplasmosis.

## Conclusion

Bovine anaplasmosis is associated with clinically relevant myocardial injury primarily driven by anaemia-induced hypoxia and compensatory tachycardia. The present study provides a comprehensive biochemical characterization of cardiac involvement in cattle with naturally occurring bovine anaplasmosis. Among the evaluated biomarkers, H-FABP and NT-proBNP demonstrated the highest diagnostic reliability for detecting myocardial injury, whereas conventional enzymes such as CK-MB showed limited diagnostic value. Routine assessment of sensitive cardiac biomarkers may therefore improve the early recognition of cardiac complications in cattle affected by anaplasmosis and support more targeted clinical decision-making in field conditions.

## Supplementary Information

Below is the link to the electronic supplementary material.

Supplementary Material 1 (DOCX 36.0 KB)

## Data Availability

All data supporting the findings of this study are available within the paper and its Supplementary Information.
