# Supplementary material for: Myocardial injury associated with naturally occurring clinical bovine anaplasmosis: evaluation of novel cardiac biomarkers
Source: Vet Res Commun. 2026 Jun 4;50(5):365. doi: 10.1007/s11259-026-11309-x (PMC13236734; doi:10.1007/s11259-026-11309-x)
Supplement: Supplementary file 1 — Supplementary Material 1 (DOCX 36.0 KB) [file 11259_2026_11309_MOESM1_ESM.docx]

**Myocardial injury associated with naturally occurring clinical bovine anaplasmosis: evaluation of novel cardiac biomarkers**

Şükrü Değirmençay^1*^, Sefa Küçükler^2^, Selçuk Özdemir^3^, Ramazan Kaman^4^, Özge Kandemir^5^, Reyhane Bayat^1^, Muhammed Kadak^6^, İrem Nur Göl^6^, Nergis Ulaş^1^, Rabia Yüksel^6^, Akın Kırbaş^1^

**RT-qPCR Assay for Hemoparasite Detection**

Previous studies have frequently reported the occurrence of *B. bigemina*, *B. bovis*, and *T. annulata* in Eastern Anatolia (Zhou et al., 2015). To investigate the potential presence of mixed infections caused by these parasites, species-specific real-time PCR (qPCR) assays targeting conserved regions of the 18S rRNA gene were employed using a TaqMan probe-based detection system. *Theileria annulata* was detected using the primers Tann-18SF (5′-AGACCTTAACCTGCTAAATAGG-3′) and Tann-18SR (5′-CATCACAGACCTGTTATTGC-3′) together with a FAM-labeled hydrolysis probe (5′-AAGTTTCTACCTGCCCGTT-3′) as previously described by Ros-García et al. (2012). Detection of *Babesia bovis* was performed using the primers bovisF (5′-ATATGTTTGCATTTGCTG-3′) and bovisR (5′-CTCCAAACCAATATGAAAG-3′), along with a species-specific VIC-labeled TaqMan probe (VIC-CAAACCATAAAGTCATCGGTATATCCTAC-MGB) (Zhang et al., 2016). *Babesia bigemina* was identified using the primers bigemF (5′-GGTCTATTTGGTGGAGTT-3′) and bigemR (5′-ACAAGACCAAATGCAATT-3′) in combination with a FAM-labeled probe (6FAM-CAATTGTTCTTGGAGCAGCT-TAMRA) (Zhang et al., 2016). All qPCR reactions were prepared in a final volume of 20 µL using TaqMan qPCR Master Mix and were performed under the following thermal cycling conditions: an initial denaturation at 95 °C for 3 min, followed by 45 cycles of 95 °C for 15 s and 60 °C for 30 s. Each sample was analysed in duplicate, and appropriate positive and no-template controls were included in all runs, with real-time fluorescence data collected during the annealing/extension step.

**References:**

Zhou M, Cao S, Sevinc F, Sevinc M, Ceylan O, Moumouni PFA, Jirapattharasate C, Liu M, Wang G, Iguchi A, Vudriko P, Suzuki H, Xuan X. Molecular detection and genetic identification of Babesia bigemina, Theileria annulata, Theileria orientalis and Anaplasma marginale in Turkey. Ticks Tick Borne Dis. 2016 Feb;7(1):126-134. doi: 10.1016/j.ttbdis.2015.09.008. Epub 2015 Sep 28. PMID: 26492823.

Ros-García A, Nicolás A, García-Pérez AL, Juste RA, Hurtado A. Development and evaluation of a real-time PCR assay for the quantitative detection of Theileria annulata in cattle. Parasit Vectors. 2012 Aug 13;5:171. doi: 10.1186/1756-3305-5-171. PMID: 22889141; PMCID: PMC3432606.

Zhang B, Sambono JL, Morgan JAT, Venus B, Rolls P, Lew-Tabor AE. An Evaluation of Quantitative PCR Assays (TaqMan® and SYBR Green) for the Detection of Babesia bigemina and Babesia bovis, and a Novel Fluorescent-ITS1-PCR Capillary Electrophoresis Method for Genotyping B. bovis Isolates. Vet Sci. 2016 Sep 13;3(3):23. doi: 10.3390/vetsci3030023. PMID: 29056732; PMCID: PMC5606575.

**Supplementary Table S1.** Real-time PCR (qPCR) results and Ct values for hemoparasitic agents in cattle

| **Group** | **Animal No** | ***B. bigemina* (Ct)** | ***B. bovis* (Ct)** | ***T. annulata* (Ct)** |
| --- | --- | --- | --- | --- |
| Anaplasmosis | 1 | Undetermined | Undetermined | Undetermined |
| Anaplasmosis | 2 | Undetermined | Undetermined | Undetermined |
| Anaplasmosis | 3 | Undetermined | Undetermined | Undetermined |
| Anaplasmosis | 4 | Undetermined | Undetermined | Undetermined |
| Anaplasmosis | 5 | Undetermined | Undetermined | Undetermined |
| Anaplasmosis | 6 | Undetermined | Undetermined | Undetermined |
| Anaplasmosis | 7 | Undetermined | Undetermined | Undetermined |
| Anaplasmosis | 8 | Undetermined | Undetermined | Undetermined |
| Anaplasmosis | 9 | Undetermined | Undetermined | Undetermined |
| Anaplasmosis | 10 | Undetermined | Undetermined | Undetermined |
| Anaplasmosis | 11 | Undetermined | Undetermined | Undetermined |
| Anaplasmosis | 12 | Undetermined | Undetermined | Undetermined |
| Healthy control | 13 | Undetermined | Undetermined | Undetermined |
| Healthy control | 14 | Undetermined | Undetermined | Undetermined |
| Healthy control | 15 | Undetermined | Undetermined | Undetermined |
| Healthy control | 16 | Undetermined | Undetermined | Undetermined |
| Healthy control | 17 | Undetermined | Undetermined | Undetermined |
| Healthy control | 18 | Undetermined | Undetermined | Undetermined |
| Healthy control | 19 | Undetermined | Undetermined | Undetermined |
| Healthy control | 20 | Undetermined | Undetermined | Undetermined |
| Healthy control | 21 | Undetermined | Undetermined | Undetermined |
| Healthy control | 22 | Undetermined | Undetermined | Undetermined |
| Healthy control | 23 | Undetermined | Undetermined | Undetermined |
| Healthy control | 24 | Undetermined | Undetermined | Undetermined |

**Supplementary Table S1 legend**

Real-time PCR (qPCR) screening results for *B. bigemina, B. bovis,* and *T. annulata* in cattle diagnosed with anaplasmosis and healthy controls. Ct values were recorded as **Undetermined** when no amplification signal was detected within 45 cycles. All samples were therefore considered negative for *Babesia* and *Theileria* species, confirming the absence of concurrent or subclinical hemoparasitic infections in the study population.

**Supplementary Table 2.** Comparison of haematological, biochemical and some clinical findings of cattle in the infected subgroups and control groups

| **Parameters** | **Unit** | **Healthy (n=12)** | **Mixed infection**  **(n=4)** | ***A. marginale* (n=4)** | ***A. centrale***  **(n=4)** | ***P* value** |
| --- | --- | --- | --- | --- | --- | --- |
| RT | °C | 38.4±0.25^A^ | 39.88±0.54^B^ | 38.83±0.76^A^ | 39.60±0.78^B^ | **<0.001** |
| RR | breaths/min | 21.7±3.17^A^ | 32±8.64^B^ | 32±5.66^B^ | 33±5.03^B^ | **<0.001** |
| HR | beats/min | 67.3±6.79^A^ | 118±7.66^B^ | 125±12.80^B^ | 110±27.23^B^ | **<0.001** |
| WBC | x10^3^/μL | 6.87 (4.26-8.04)^A^ | 12.2 (8.18-29.4)^B^ | 11.3 (8.18-16.9)^B^ | 9.24 (6.53-11.7)^A^ | **0.002** |
| Lymphocyte count | x10^3^/μL | 3.95 (2.68-4.79)^A^ | 6.52 (5.77-14.6)^B^ | 7.26 (6.54-8.08)^B^ | 4.03 (3.79-8.71)^A^ | **0.005** |
| Monocyte count | x10^3^/μL | 0.26±0.11^A^ | 0.43±0.13^AB^ | 0.53±0.20^B^ | 0.27±0.19^A^ | **0.018** |
| Neutrophil count | x10^3^/μL | 2.28±0.89^A^ | 6.63±5.34^B^ | 4.02±3.75^AB^ | 3.62±2.38^AB^ | 0.086 |
| Eosinophil count | x10^3^/μL | 0.10 (0.03-0.25) | 0.09 (0.05-0.15) | 0.06 (0.04-0.11) | 0.07 (0.05-0.39) | 0.809 |
| RBC | x10^6^/μL | 5.89±0.45^B^ | 2.91±1.38^A^ | 3.82±1.01^A^ | 7.15±1.01^C^ | **0.010** |
| HGB | g/dL | 8.48±0.76^B^ | 4.43±1.37^A^ | 5.3±0.94^A^ | 9.3±1.63^B^ | **0.002** |
| HCT | % | 25.4±2.65^B^ | 11.76±3.65^A^ | 14.7±3.78^A^ | 25.3±4.41^B^ | **<0.001** |
| MCV | fL | 44.5 (37-48)^A^ | 37.5 (36-66)^A^ | 38 (31-49)^A^ | 35.5 (32-37)^B^ | **0.048** |
| MCH | pg | 14.4 (12.8-15.4) | 14.9 (12.1-25.4) | 13.4 (12.9-17.3) | 12.5 (12.6-14.6) | 0.233 |
| MCHc | g/dL | 33.5±1.19^A^ | 37.8±4.43^B^ | 36.9±4.28^AB^ | 36.9±2.79^AB^ | **0.031** |
| RDW | % | 23.2±2.20^A^ | 27.8±4.27^B^ | 27.48±1.12^B^ | 26.5±2.63^AB^ | **0.009** |
| PLT | x10^3^/μL | 243 (38-981) | 365 (205-613) | 386 (320-420) | 272 (97-414) | 0.205 |
| H-FABP | ng/mL | 0.43±0.06^A^ | 0.66±0.04^B^ | 0.63±0.04^B^ | 0.61±0.07^B^ | **<0.001** |
| NT-proBNP | ng/mL | 0.21±0.06^A^ | 0.41±0.05^B^ | 0.34±0.08^B^ | 0.33±0.13^B^ | **0.004** |
| cTnI | ng/mL | 0.06 (0.04-0.07)^A^ | 0.12 (0.07-0.19)^B^ | 0.07 (0.03-0.16)^AB^ | 0.12 (0.05-0.34)^AB^ | **0.017** |
| CK-MB | U/L | 119±21.6 | 166±78.7 | 179±60.8 | 134±31.8 | 0.090 |
| CK | U/L | 187±73.2^A^ | 1190±691^B^ | 2141±773^C^ | 249±255^A^ | **<0.001** |
| LDH | U/L | 937±193^A^ | 2042±169^B^ | 2360±463^B^ | 1220±324^A^ | **<0.001** |
| AST | U/L | 55.4±6.79^A^ | 182±78^B^ | 198±50.5^B^ | 88±50.4^A^ | **<0.001** |
| ALT | U/L | 31.7±10 | 23.2±4.19 | 39±5.62 | 29.8±10.6 | 0.128 |
| ALP | U/L | 49 (17-268) | 91 (45-112) | 98.5 (62-135) | 89.5 (55-148) | 0.310 |
| GGT | U/L | 11.9±4.60^A^ | 26±17.2^AB^ | 30±21.6^B^ | 14.3±5.68^AB^ | 0.038 |
| TBIL | mg/dL | 0.02 (0.01-0.06)^A^ | 1.07 (0.72-3.30)^B^ | 0.320 (0.14-1.24)^A^ | 0.105 (0.01-0.23)^A^ | **0.001** |
| DBIL | mg/dL | 0.02 (0.01-0.03) | 0.41 (0-0.67) | 0.12 (0.01-0.24) | 0.07 (0.03-0.11) | 0.134 |
| TP | g/dL | 6.20±1.19 | 6.31±1.03 | 6.50±0.271 | 5.60±0.708 | 0.628 |
| ALB | g/dL | 2.57±0.48 | 2.24±0.39 | 2.38±0.22 | 2.34±0.45 | 0.528 |
| BUN | mg/dL | 10.9 (9.84-15.8)^A^ | 35.1 (18.6-48.3)^B^ | 38.3 (28.9-76.2)^B^ | 20.7 (12.4-46.7)^B^ | **<0.001** |
| Cr | mg/dL | 0.91 (0.79-1.14) | 0.75 (0.53-0.97) | 0.83 (0.22-4.35) | 1.03 (0.9-1.78) | 0.285 |
| Glucose | mg/dL | 61.1±12.1^A^ | 58.5±7.51^A^ | 81.5±11^B^ | 68.3±11.3^AB^ | **0.024** |

RT: Rectal temperature; HR: Heart rate (per min); RR:Respiratory rate (per min); WBC: white blood cell; RBC: red blood cell; HGB: hemoglobin; HCT: hematocrit; MCV: mean erythrocyte volume; MCH: mean erythrocyte hemoglobin; MCHC: mean erythrocyte hemoglobin concentration; RDW: erythrocyte distribution width; PLT: platelet; H-FABP: heart type fatty acid binding protein; NT-proBNP: N-terminal pro-peptide natriuretic type B; cTnI: cardiac troponin I; CK-MB; creatine kinase myocardial band; CK; creatine kinase; LDH: lactate dehydrogenase; AST: aspartate aminotransferase; ALT: alanine aminotransferase; ALP: alkaline phosphatase; GGT: gamma glutamyl transferase; TBIL: total bilirubin; DBIL: direct bilirubin; TP: total protein; ALB: albumin; BUN: blood urea nitrogen; Cr: creatinine; Parametric data are expressed as mean±SD, and nonparametric data are expressed as median (min–max). Different superscript letters (A, B, AB) within the same row indicate statistically significant differences between groups (P < 0.05). Groups sharing at least one common letter are not significantly different.
